# Effect of High-Temperature Nitridation and Buffer Layer on Semi-Polar (10–13) AlN Grown on Sapphire by HVPE

**DOI:** 10.3390/mi12101153

**Published:** 2021-09-25

**Authors:** Qian Zhang, Xu Li, Jianyun Zhao, Zhifei Sun, Yong Lu, Ting Liu, Jicai Zhang

**Affiliations:** 1 College of Mathematics and Physics, Beijing University of Chemical Technology, Beijing 100029, China; zhang2020@buct.edu.cn (Q.Z.); xuli@mail.buct.edu.cn (X.L.); jyzhao@mail.buct.edu.cn (J.Z.); luy@mail.buct.edu.cn (Y.L.); 2 School of Physical Education and Health Management, Guangxi Normal University, Guilin 541001, China; szfszfffbb@163.com; 3 State Key Laboratory of Chemical Resource Engineering, Beijing University of Chemical Technology, Beijing 100029, China

**Keywords:** HVPE, AlN, high-temperature, buffer layer, nitridation

## Abstract

We have investigated the effect of high-temperature nitridation and buffer layer on the semi-polar aluminum nitride (AlN) films grown on sapphire by hydride vapor phase epitaxy (HVPE). It is found the high-temperature nitridation and buffer layer at 1300 °C are favorable for the formation of single (10–13) AlN film. Furthermore, the compressive stress of the (10–13) single-oriented AlN film is smaller than polycrystalline samples which have the low-temperature nitridation layer and buffer layer. On the one hand, the improvement of (10–13) AlN crystalline quality is possibly due to the high-temperature nitridation that promotes the coalescence of crystal grains. On the other hand, as the temperature of nitridation and buffer layer increases, the contents of N-Al-O and Al-O bonds in the AlN film are significantly reduced, resulting in an increase in the proportion of Al-N bonds.

## 1. Introduction

AlN is a potential material for the deep ultraviolet (DUV) optical devices, such as light-emitting diodes (LEDs) and laser diodes (LDs) [1], since it has wide direct band gap of 6.2 eV and excellent physical and chemical stability. These optoelectronic devices are generally grown along AlN c-axis [2,3]. However, the strong spontaneous and piezoelectric polarization along c-axis will weaken the recombination of carriers in the quantum wells [4], which in turn decrease the luminescence efficiency of devices. The use of semi-polar substrates and epitaxial layers [5,6,7,8,9], such as (10–11), (10–12) and (10–13) AlN, can effectively solve this problem since the polarization field is greatly weakened [10,11]. According to the energy band structure, other semi-polar plane, such as (11–22), can produce a negative polarization field across multiple quantum wells used in optoelectronic devices, which can reduce the confinement of hole states and increase the carrier loss. In contrast, the (10–13) plane has a positive polarization field, which is favorable for the devices [12].

Semi-polar nitride films were generally grown on M-plane sapphire [5,6], silicon [7,8] and ZnO [13,14]. It is also reported that native semi-polar AlN substrate were used for homoepitaxial semi-polar AlN [15]. Shen et al. obtained high quality (10–13) AlN by ammonia-free metalorganic vapor phase at high temperature of 1650 °C [5]. Kukushkin er al. investigated the semi-polar AlN without cracks on Si (001) and hybrid SiC/Si (001) substrates [7]. Bessolov et al. prepared the hexagonal AlN layer on Si with V-groove nanostructured surface by HVPE at 1080 °C [8]. Ueno et al. obtained high quality semi-polar AlN and AlGaN on ZnO substrates with annealing in the air by growing a room temperature epitaxial AlN buffer layer [13,14].

Due to the transparency and low cost, sapphire substrate is normally used for preparation of AlN substrates and AlN-based deep ultraviolet optoelectronic devices. For (10–13) AlN on m-plane sapphire, the in-plane epitaxial relationship between (10–13) AlN and m-plane sapphire is [30-3-2]_AlN_//[1–210]_sapphire_ and [1–210]AlN//[0001]sa_pphire_ [5]. According to the epitaxial relationship, it can be determined that the lattice mismatch between m-plane sapphire and (10–13) AlN is the smallest [12]. When growing AlN on sapphire [16,17,18,19], nitridation is a common method to improve crystal quality [20,21]. Moreover, nitridation has a large impact on the crystal orientation of semi-polar AlN [22,23]. Furthermore, it is well known that buffer layer is an efficient approach to reduce the lattice mismatch between III-V nitrides and foreign substrates [24]. At present, the effect of the buffer layer on the quality of polar AlN crystals has been widely confirmed [24,25,26,27]. In addition, compared with metalorganic chemical vapor deposition (MOCVD), hydride vapor phase epitaxy (HVPE) is more suitable for AlN substrate due to the high growth rate and low cost [28]. However, the comprehensive influence of high-temperature nitridation and high-temperature buffer layer on the semi-polar AlN film grown by HVPE has not yet been clarified.

In this work, we use HVPE to grow semi-polar AlN on m-plane sapphire substrate. The influence of high-temperature nitridation and buffer layer on the semi-polar AlN films has been carefully studied.

## 2. Experiment

The growth of semi-polar (10–13) AlN sample was performed in a home-made horizontal HVPE system. 2-inch m-plane sapphire with miscut-angle of ±0.1° was used as substrate, and HCl and NH_3_ were used as input active gases. The mixture of H_2_ and N_2_ were utilized as carrier gas under 40 Torr. At first, the m-plane sapphire substrate was heated to the nitridation temperature in the carrier gas and kept for 10 min in H_2_ ambient to remove the surface pollution and achieve thermal stability. The sapphire substrate was then nitrided in NH_3_ ambient about 10 min. Next, the buffer layer was grown for 1 min. Finally, the temperature was raised to 1500 °C to grow AlN film for 30 min. Table 1 shows the growth conditions of four samples with different nitridation temperature and buffer layer temperature. X-ray diffractometer (XRD, Philips, X’pert MRD PIXcel, Amsterdam, The Netherlands) was used to characterize the orientation and quality of AlN film. Scanning electron microscope (SEM, HITACHI, SU8020, Tokyo, Japan) was used to study the cross-section morphology. Raman spectrometer (HORIBA, LabRam HR Evolution, Kyoto, Japan) was used to study the stress distribution of AlN film. X-ray photoelectron spectroscopy (XPS, Thermo Scientific, Escalab 250Xi, Waltham, MA, USA) was used to analyze the chemical composition of the AlN film.

## 3. Results and Discussion

**Figure 1:**
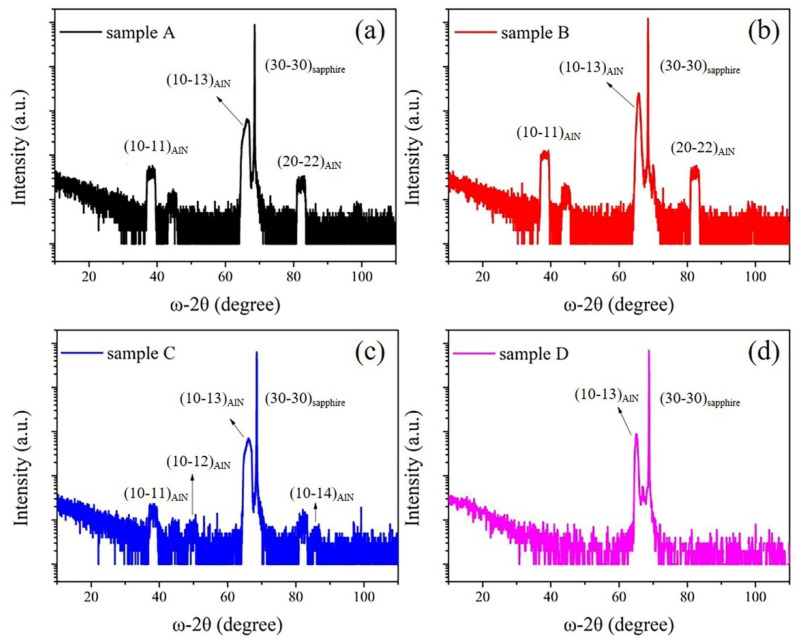
(**a**–**d**) corresponding to XRD ω−2θ scan of sample A, sample B, sample C and sample D, respectively.

Nitridation and buffer layer are introduced to grow semi-polar (10–13) AlN by HVPE. As shown in Figure 1, the growth orientation of the semi-polar AlN films is characterized by XRD ω-2θ scan. Low-temperature (1050 °C) nitridation and low-temperature (800 °C) buffer layer are first tried to grow semi-polar (10–13) AlN. In Figure 1a, (10–11) and (20–22) diffraction peaks are also observed except the strong (10–13) diffraction peak, indicating that sample A is not the single crystal. In sample B, the buffer layer temperature is kept at 800 °C and the nitridation temperature is increased to 1300 °C. Although the additional diffraction peaks still exist, the (10–13) diffraction peak is narrowed as shown in Figure 1b. In contrast, for sample C, the nitridation temperature is 1050 °C, while the buffer layer temperature rises to 1300 °C. From Figure 1c, it is clearly to see that the intensity of impurity peaks of (10–11) and (20–22) is significantly reduced. Comparing with sample A, B and C, we can conclude that high-temperature nitridation and high-temperature buffer layer are promising to improve the crystal quality of the semi-polar (10–13) AlN. Therefore, both high-temperature (1300 °C) nitridation and high-temperature buffer (1300 °C) layer are used in sample D. As expected, in Figure 1d, there are no other impurity peaks but only sharp (10–13) AlN diffraction peak, implying that sample D is (10–13)-oriented single crystal. The weak peak between (10–13) AlN and (30–30) sapphire is most probably a shoulder peak due to the twin structures, which are common small-angle grains in (10–13) AlN [29]. Compared with sample D, an additional broad peak around 44.4° exits in samples A-C. Unfortunately, the origin of the peak is unknown currently. But from the characterizations of this peak, we believe it should come from AlN.

**Figure 2:**
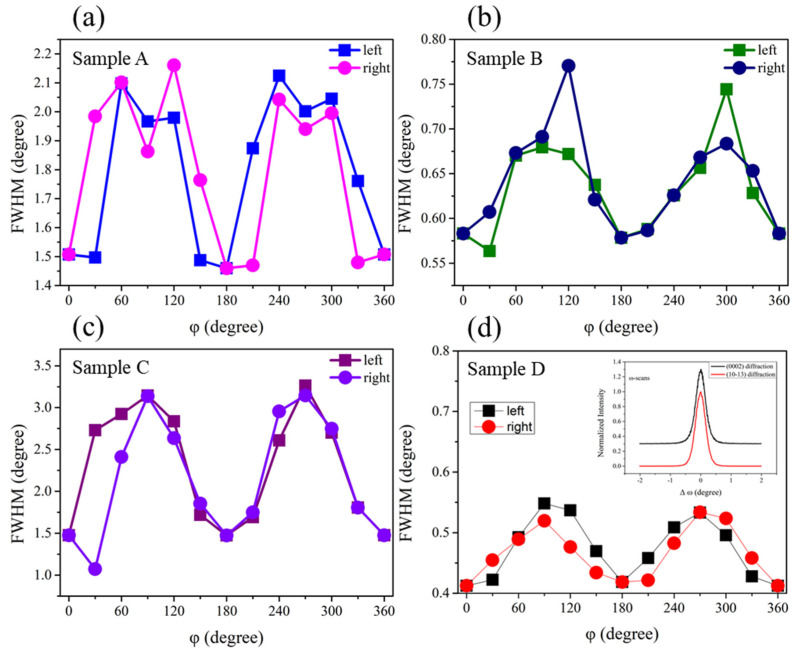
(**a**–**d**) is the variations of FWHM values of XRC at different φ angle for (10–13) AlN films of sample A, sample B, sample C and sample D, respectively. The left and right corresponding to different FWHM values of XRC at the same φ angle. The insert of figure (**d**) shows the FWHM of (0002) and (10–13) diffraction.

Figure 2 shows the variations of full width at half maximum (FWHM) of (10–13) ω scans at different angle ϕ in the range of 0° and 360° for sample A, B, C and D, respectively. There are two FWHM values at the same φ angle due to the effect of stress. It can be clearly observed that the change of FWHM has an M-shaped curve, indicating that it has obvious anisotropy characteristics. The FWHM value of sample B is significantly lower than that of sample A, suggesting that the crystal quality has been significantly improved after increasing the temperature of the buffer layer. All the FWHM values for sample D are smaller than those of other samples, demonstrating that high-temperature nitridation and high-temperature buffer layer can improve the crystal quality of (10–13) AlN films significantly. The insert of Figure 2d shows the rocking curves of (0002) and (10–13) diffraction. The FWHM values are 0.359° and 0.356° for (0002) and (10–13) diffraction, respectively. It is very close to the value of literature [6].

**Figure 3:**
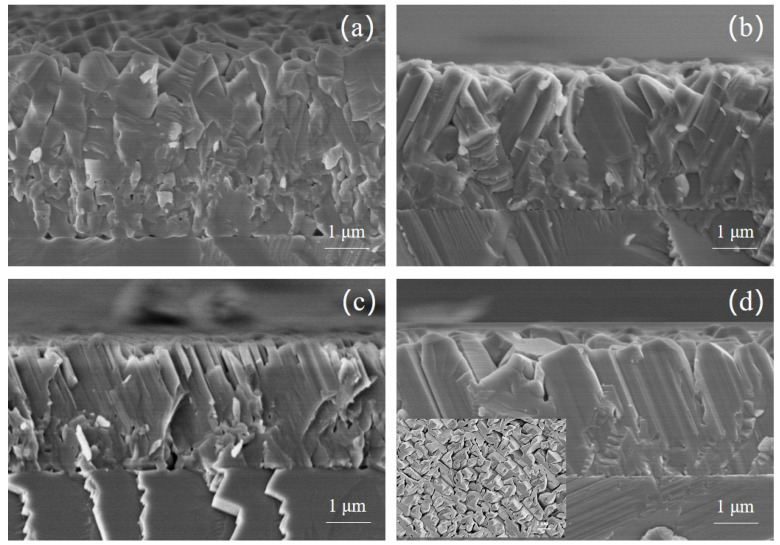
(**a**–**d**) corresponding to the cross-sectional SEM image of sample A, sample B, sample C and sample D, respectively. The insert of figure (**d**) shows the surface SEM image of sample D, in which the scale bar is 1 um.

Figure 3 shows the cross-sectional SEM images of the four samples. In sample D (Figure 3d), the crystal column extends from the growth interface to the surface, and the orientation of the crystal column is almost the same, which is consistent with the result of XRD. However, the crystal columns are still not coalescent, as shown in the insert of Figure 3d. While in the other three samples, the crystal columns become smaller and their orientation looks disordered. Moreover, in sample A, there are many holes as large as 200 nm at the growth interface. In the case of low-temperature nitridation and buffer layer, high density of AlN defects appear and AlN grains are not easy to merge at the initial growth stage [6], resulting in that sapphire can’t be completely covered by AlN film. During the high-temperature (1500 °C) growth, due to lack of AlN protective layer, the exposed sapphire surface is decomposed, leading to large holes in sample A as shown in Figure 1a.

**Figure 4:**
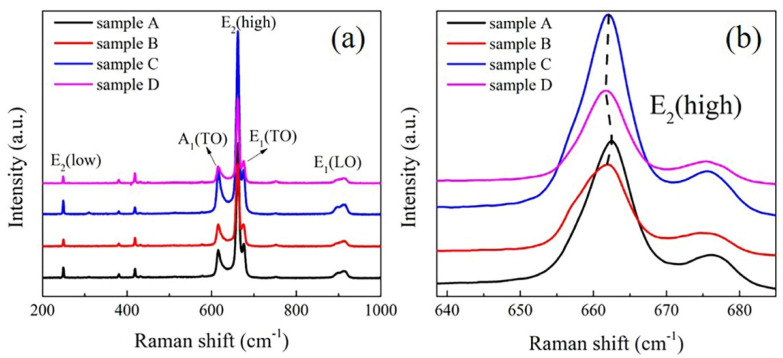
(**a**) Raman spectra of AlN samples, including E_2_(low), A_1_(TO), E_2_(high), E_1_(TO) and E_1_(LO) peaks of AlN. (**b**) The enlarged view of the E_2_(high) peaks in (**a**). The black, red, blue and pink curves corresponding to sample A, B, C and D, respectively. The black dash line is guide for eyes.

Raman spectrum is then introduced to evaluate the residual stress in the semi-polar AlN films. In the x(yy)z scattering configuration [30], E_2_(low), A_1_(TO), E_2_(high), E_1_(TO) and E_1_(LO) Raman peaks of AlN can be observed in Figure 4a. Among these peaks, E_2_(high) peak could reflect the residual stress of AlN film and the value of E_2_(high) peak under stress free is 657.4 cm^−1^ [7]. The enlarged image (Figure 4b) of E_2_(high) peak demonstrates that the peak values of the four samples are larger than 657.4 cm^−1^, which means they are all in the compressive state. Furthermore, the E_2_(high) peak value of sample B and D is smaller than A and C, indicating that the buffer layer at high temperature is beneficial to reduce the compressive stress of (10–13) AlN film. 

**Figure 5:**
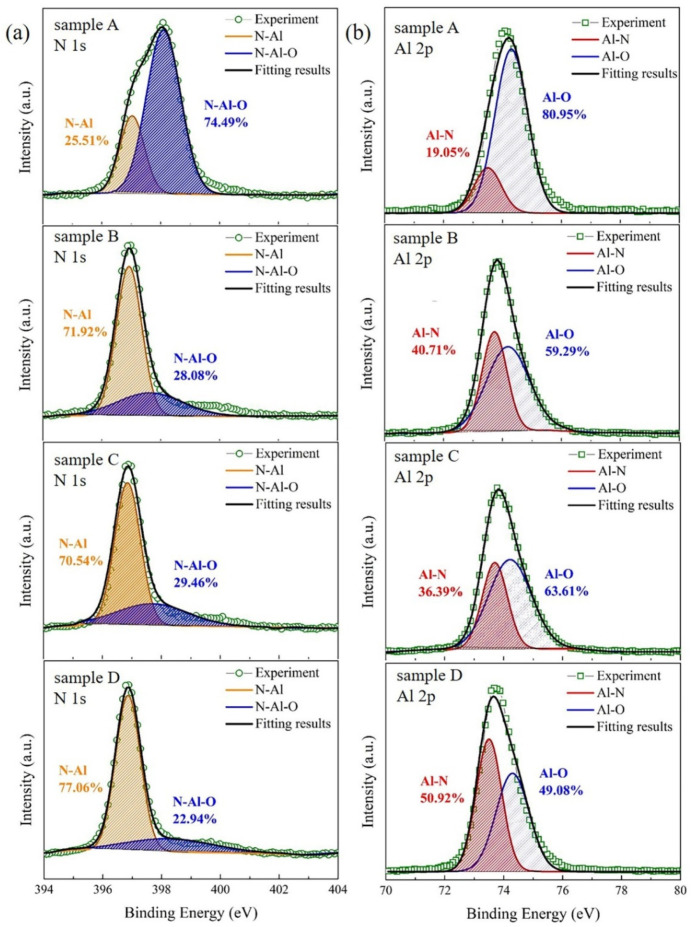
(**a**) N 1s high-resolution XPS scans of sample A, B, C and D. The green circle, orange, blue and black curves corresponding to the experiment result, N−Al bond, N−Al−O bond and fitting result, respectively. (**b**) Al 2p high-resolution XPS scans of sample A, B, C and D. The green square, red, blue and black curves corresponding to the experiment result, Al−N bond, Al−O bond and fitting result, respectively.

In order to investigate the effect of nitridation and buffer layer on the chemical states of AlN films, we perform an XPS core level measurement. Figure 5 shows the XPS core level spectra of N 1s and Al 2p. In Figure 5a, the N 1s spectrum in all samples can be deconvoluted into two peaks at 396.8 ± 0.2 eV and 397.7 ± 0.3 eV, corresponding to the N−Al bond and N−Al−O bond, respectively [31]. In sample A, the relative area of the N−Al peak is only 25.51% while the relative area of the N−Al−O peak is as large as 74.49%. With the increasing temperature of nitridation and buffer layer, the relative area of the N−Al peak is enhanced obviously, increasing to 77.06% in sample D. As a result, comparing with sample A, the center of the N 1s spectra shifts from 398.1 eV to 396.7 eV in sample D due to the expanded content of N−Al peak [31]. Furthermore, in Figure 5b, the Al−N and Al−O peaks in the Al 2p spectrum are centered at 73.5 ± 0.2 eV and 74.3 ± 0.1 eV, respectively [21]. It is obvious that sample D has the larger Al−N content and the smaller Al−O content than other samples. Therefore, both N 1s and Al 2p spectrum show that the high-temperature nitridation and buffer layer are beneficial to reduce the combination of oxygen impurity with Al atom or N atom, resulting in a better AlN film.

## 4. Conclusions

In conclusion, with the high-temperature nitridation and buffer layer, the single (10–13) AlN film is successfully obtained on m-plane sapphire by HVPE. Comparing with the polycrystalline samples which have the low-temperature nitridation layer and buffer layer, there is the smallest compressive stress in the (10–13) single-oriented AlN film. The simultaneous introduction of high-temperature nitridation and buffer layer is beneficial to promote the coalescence of crystal grains and reduce the content of impurity components, like N−Al−O and Al−O, thus improving the crystal quality of semi-polar (10–13) AlN.

## Figures and Tables

**Table 1:** Growth conditions of sample A, B, C and D.

Sample	Nitridation Temperature/°C	Temperature of Buffer Layer/°C	Growth Temperature/°C
A	1050	800	1500
B	1050	1300	1500
C	1300	800	1500
D	1300	1300	1500

## Data Availability

Data sharing is not applicable to this article.
